# Scratching the Itch: Updated Perspectives on the Schistosomes Responsible for Swimmer’s Itch around the World

**DOI:** 10.3390/pathogens11050587

**Published:** 2022-05-16

**Authors:** Eric S. Loker, Randall J. DeJong, Sara V. Brant

**Affiliations:** 1 Center for Evolutionary and Theoretical Immunology, Parasites Division, Museum of Southwestern Biology, Department of Biology, University of New Mexico, Albuquerque, NM 87131, USA; sbrant@unm.edu; 2 Department of Biology, Calvin University, Grand Rapids, MI 49546, USA; rdejong@calvin.edu

**Keywords:** swimmer’s itch, cercarial dermatitis, schistosomiasis, zoonosis, One Health

## Abstract

Although most studies of digenetic trematodes of the family Schistosomatidae dwell on representatives causing human schistosomiasis, the majority of the 130 identified species of schistosomes infect birds or non-human mammals. The cercariae of many of these species can cause swimmer’s itch when they penetrate human skin. Recent years have witnessed a dramatic increase in our understanding of schistosome diversity, now encompassing 17 genera with eight more lineages awaiting description. Collectively, schistosomes exploit 16 families of caenogastropod or heterobranch gastropod intermediate hosts. Basal lineages today are found in marine gastropods and birds, but subsequent diversification has largely taken place in freshwater, with some reversions to marine habitats. It seems increasingly likely that schistosomes have on two separate occasions colonized mammals. Swimmer’s itch is a complex zoonotic disease manifested through several different routes of transmission involving a diversity of different host species. Swimmer’s itch also exemplifies the value of adopting the One Health perspective in understanding disease transmission and abundance because the schistosomes involved have complex life cycles that interface with numerous species and abiotic components of their aquatic environments. Given the progress made in revealing their diversity and biology, and the wealth of questions posed by itch-causing schistosomes, they provide excellent models for implementation of long-term interdisciplinary studies focused on issues pertinent to disease ecology, the One Health paradigm, and the impacts of climate change, biological invasions and other environmental perturbations.

## 1. Introduction

**Figure 1:**
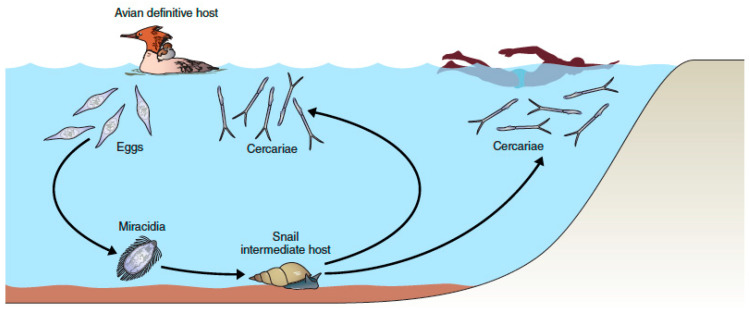
Typical life cycle of an avian schistosome. In this case, *Trichobilharzia stagnicolae* is commonly implicated in swimmer’s itch outbreaks in oligotrophic lakes in Michigan, in the northern USA. Note the involvement of an avian definitive host such as the common merganser (*Mergus merganser*) in which adult worms mate and reproduce, resulting in discharge of schistosome eggs into the water. Eggs hatch and release swimming, ciliated miracidia that locate and penetrate the freshwater snail host *Stagnicola emarginata*. A miracidium transforms into a mother sporocyst that produces multiple daughter sporocysts that migrate to the snail’s digestive gland where they produce numerous cercariae. The cercariae exit the snail, swim, and are carried by currents or wave action, and once they have located a merganser will penetrate the skin and continue the life cycle. People in contact with the water are also at risk of skin penetration by the cercariae, which typically incite a strong inflammatory reaction, swimmer’s itch, and usually, but not always, die in the skin.

Members of the digenetic trematode family Schistosomatidae are unusual trematodes in having separate adult male and female worms that inhabit the vascular systems of their avian or mammalian definitive hosts. Their generalized life cycle (Figure 1) is typical of trematodes in that they must undergo an obligatory period of larval development in molluscs, in this case particular species of freshwater or marine gastropods. One distinctive aspect of the schistosome life cycle germane to this volume on avian schistosomes is that the free-swimming cercariae that are produced in large numbers in their snail hosts, once released into water do not encyst in or on other hosts and await ingestion by the definitive host, as is typical of many trematode life cycles. Rather, schistosome cercariae penetrate directly into the skin or other epithelial surfaces, such as in the mouth or throat, to gain entrance into their definitive hosts. Cercariae are highly adapted for their role in locating hosts and then penetrating the skin barrier [1,2,3]. Once in their normal definitive host, they undertake a complex migration route to the intravascular system before they attain sexual maturity and produce eggs, which are passed in the feces, or occasionally by other routes. Insofar as schistosome species that normally develop in birds and non-human mammals can penetrate human skin and cause varying degrees of pathology, the phenomenon of swimmer’s itch exemplifies a zoonosis, i.e., a disease transmissible from animals to humans. 

Schistosomes achieve their greatest notoriety for their role in causing the neglected tropical disease of human schistosomiasis. Three species (*Schistosoma mansoni, S. haematobium* and *S. japonicum*) are primarily responsible for causing the 220 million cases of schistosomiasis, with a few additional species (*S. mekongi, S. intercalatum* and *S. guineensis*) playing secondary roles [4].

Often overlooked is the fact that these six human-infective *Schistosoma* species comprise only a small minority of species in the Schistosomatidae [5]. There are 20 additional named species in *Schistosoma* that parasitize a variety of mammals depending on the species (often ruminants, pigs, dogs, rodents or even hippos). There are three additional genera of mammalian schistosomes, *Bivitellobilharzia* with two species known from elephants primarily and Asian rhinos, and the monotypic North American endemic genera *Schistosomatium* of rodents, and *Heterobilharzia* of raccoons and other mammals. The remaining 85 species, comprising 13 named genera, are all parasites of birds. No known schistosome species can mature in both birds and mammals, and no known hosts for members of the Schistosomatidae are found in non-endothermic hosts. *Griphobilharzia amoena*, a blood fluke of freshwater crocodiles in Australia, was originally considered to be a schistosome, but molecular phylogenetic studies suggest it is an unusual member of the related family Spirorchiidae [6]. 

Each schistosome species relies on particular species or genera of gastropods as intermediate hosts. They are relatively host specific, with some species developing in particular lineages of freshwater gastropods, and others in particular lineages of marine gastropods. No known schistosome exploits both marine and freshwater gastropods or is known to be dependent on fully terrestrial gastropods, though some exploit amphibious snail hosts. Without the appropriate species of both bird or mammal definitive hosts and the requisite specific species of gastropod hosts required, the schistosome species involved would perish.

As we and others have pointed out, these many non-human schistosome species are of considerable biological interest in their own right, but they often attract attention because of the ability of their cercariae to penetrate human skin, in some cases resulting in only a small papule, but often inciting a strong allergic immune response known as cercarial dermatitis, i.e., swimmer’s itch or schistosome dermatitis. Mild forms of dermatitis can occur following primary exposure to human schistosomes as well, as shown by recent controlled experimental exposures to *S. mansoni* involving human volunteers [7], and more severe dermatitis may occur in some people upon repeated exposures to cercariae of human schistosomes [8]. However, it is the non-human schistosomes that are usually implicated in causing swimmer’s itch. Naïve individuals usually develop small but still observable papules [9,10], and repeated exposure frequently results in an increase in papule size and intensity of the response [10]. Furthermore, it has been argued that the severe inflammatory reactions developing in the skin following repeated exposure to cercariae of non-human schistosomes represent a protective response that prevents parasites from reaching the lungs or central nervous system and causing more severe damage [11]. 

Swimmer’s itch can potentially occur worldwide whenever people contact the water in freshwater or marine habitats where gastropod species occur that serve as the essential intermediate hosts for the schistosome species present. The preponderance of literature on swimmer’s itch derives from wealthier countries where itch outbreaks are common in recreational areas in warm months and are viewed as an unfortunate by-product of a summer holiday. However, in poorer countries where people often engage in labor-intense activities such as manual rice planting or tending of domestic animals that involve water contact, swimmer’s itch can be a persistent and unpleasant burden, one that often falls disproportionately on children and women [12]. Epidemiological studies to gauge the prevalence and health costs of swimmer’s itch are relatively few and are confined to developed countries [13,14,15,16]. A significant but rarely quantified cost of swimmer’s itch is borne by the summer recreation industry, especially around freshwater lakes popular for swimming [17].

The purpose of this review is to provide an overview of our growing understanding of schistosome diversity, highlighting the expanding variety of species and distinct kinds of transmission pathways known to occur. The review discusses our increasing appreciation for the complex web of ecological interactions in which itch-causing schistosome species are enmeshed. Swimmer’s itch, with its involvement of zoonotic parasites that infect wild and domestic vertebrates and humans, provides an excellent example of the value of the One Health perspective to achieve a full appreciation of the involvement of itch-causing schistosomes in natural and man-made settings [18,19,20]. We discuss some of the pros and cons of new methods for identifying where itch-causing schistosomes occur, and how we might proceed to mitigate their effects on human well-being. We then identify areas where we are still in need of more concrete information to attain a fuller understanding of the dynamics of swimmer’s itch transmission and conclude with observations about the orphan status swimmer’s itch has had with respect to funding, and how this might be overcome. 

Thanks to the availability of new tools and to the hard work of a new generation of investigators, the study of itch-causing schistosomes has had a renaissance in recent years, and the prospects for more exciting results are very bright. This effort is especially timely given the current tenuous status of much of the world’s biodiversity and impending changes due to species introductions and climate warming, to cite just two of several factors likely to change the composition of the world’s schistosome fauna. 

## 2. A Growing Appreciation for the Full Extent of Schistosome Diversity

The study of schistosome diversity has long been impeded by a number of factors. Particularly for avian schistosomes, because of their long thread-like bodies and intravascular habits, it is not trivial to find or retrieve intact adult worms, or even portions of worms. This in many cases has complicated attempts to provide adequate descriptions of adult worms upon which species descriptions and delineations are made. The results, though invaluable in many respects, are an oft-confusing legacy of species names and descriptions that are hard to relate to modern investigations. Additionally, it is becoming increasingly difficult to obtain permits to obtain and necropsy wild definitive or intermediate hosts, especially as some host species become rare. Collection of rare definitive hosts might also lead, unwittingly, to the extinction of the very parasites one might be interested in studying [21]. Critically needed life cycle studies, involving experimental infections of definitive hosts, require approvals by animal care and use committees. It is very difficult to lab-rear and maintain some of the key definitive hosts most in need of study. Coupled with the need to maintain supportive snail colonies, lab-based studies of avian schistosomes can be difficult and expensive. 

**Figure 2:**
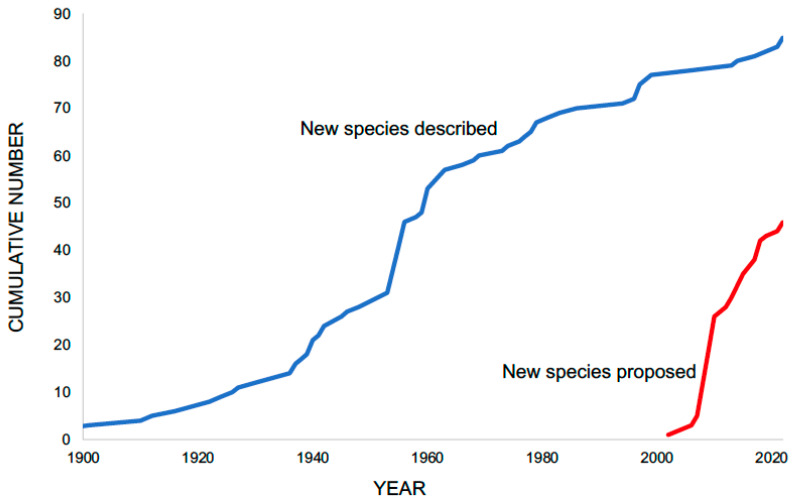
Sequence-based identification of new avian schistosome lineages. The blue line represents avian schistosome species formally described over time. The red line identifies the number of new, distinct lineages of schistosomes identified from molecular signatures, many from cercariae derived from field-collected snails.

Nonetheless, in spite of these difficulties, the overall picture of the diversity of nonhuman schistosomes has clarified considerably. This, in large measure, is due to the application of molecular methods that have enabled unambiguous detection of several new lineages over the past 20 years (Figure 2), detection based on objective reference sequences obtained from what might have once been an anonymous piece of an adult worm [22], or from a few schistosome eggs [23]. To further this quest, along with molecular information, Blasco-Costa et al. [24] emphasized the value of a holistic approach using what available information could be obtained from adult anatomy, life cycle stages and other sources of information. Extensive sampling of natural snail populations has resulted in the collection of thousands of samples of schistosome cercariae that provide reference points not only for their distinctive size, behavior and basic morphological features, but as a source of sequence data [25]. Reference cercariae sequences in a growing database enable direct comparison with sequences from adult schistosomes or eggs obtained by investigators who may be far removed in time and space from the collectors of the cercariae from snails. Recent papers indicate that this has happened on multiple occasions [22,26,27,28,29].

Another historical hindrance to making progress in defining the basic players in schistosome life cycles has been in grappling with identifications of the schistosome-infected snail hosts recovered. Although gastropod identifications and phylogenetics are far from being fully resolved matters [30,31,32], enormous progress has been made in the last couple of decades in unsnarling gastropod species identifications and providing much more reliable classification schemes than available to parasitologists in the past [33,34,35,36,37,38,39]. Just as with the schistosomes themselves, provision of reference sequences for infected gastropods has helped steadily build a more complete and reliable picture of schistosome-snail relationships. 

An important third leg in this triad of improvements is the heightened awareness of the need to submit voucher specimens (for both future morphological and molecular study) of schistosomes and, equally significant, their definitive or intermediate hosts, to natural history collections [24,40,41]. Such collections can both provide long-term curation of the specimens and integrative databases relating parasite species directly to the host species from which they were originally retrieved. But most importantly they provide a way to resample the past with new methods, resources, questions, or just to compare the past and present. This can be particularly relevant for epidemiological studies where recording change over time is critical. Most genetic studies, and many morphology studies, do not have voucher material that can be reexamined and verified. Needless to say, the value of natural history collections including genomics resources cannot be overstated in a world experiencing an ongoing extinction crisis (see Brant et al., in this volume).

**Figure 3:**
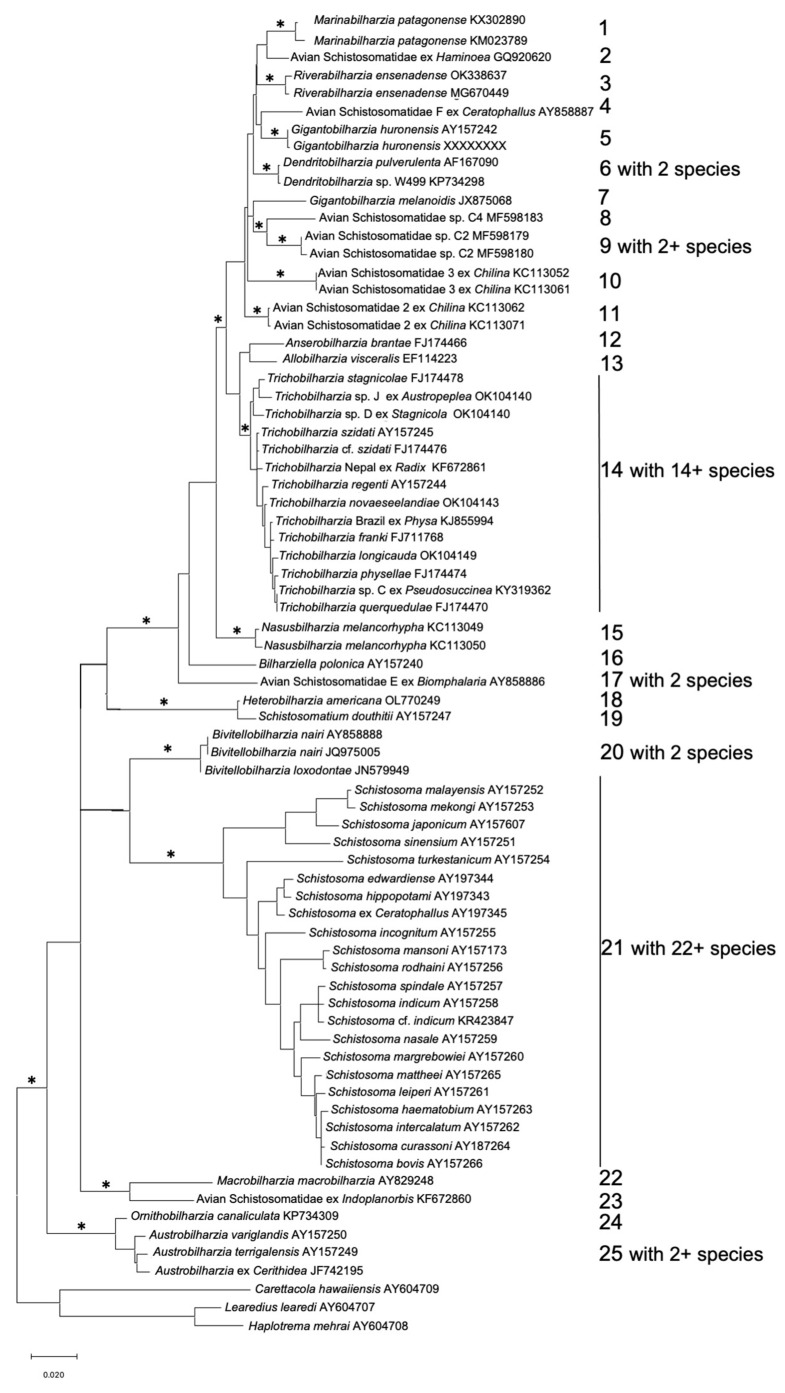
Overview of relationships among members of the Schistosomatidae based on published ~1200 bp of 28S sequence. On the right, numbered sequentially from the top, are shown 25 putative genus-level lineages, 17 of described genera (including *Marinabilharzia* and *Riverabilharzia* recently described) and 8 additional probable generic-level lineages. Indicated on the right, also, are conservative numbers of species for the speciose genera. For the avian schistosomes, preliminary sequence data suggest at least 12 additional species remain to be described. Asterisks indicate Bayesian posterior probabilities at >0.95.

An overview of the phylogenetic relationships among lineages of schistosomes is shown in Figure 3 and highlights the advances in documenting species diversity. The sequential numbers on the right side of the tree are indicative of lineages relatable to generic level differences. Note that several of the indicated genera are well established (such as *Austrobilharzia*, *Schistosoma* or *Trichobilharzia*), some have been relatively recently described (such as *Nasusbilharzia*, *Marinabilharzia* or *Riverabilharzia*), and some likely new genera await additional study. Other lineages are known but not shown here for lack of comparable amounts of sequence information. The basic features of the tree, as highlighted in several papers [22,42,43,44,45] (Ebbs et al., this volume), are: (1) a basal clade of avian schistosomes dependent on marine snails (*Austrobilharzia*, *Ornithobilharzia*); (2) an enigmatic and poorly supported lineage of avian schistosomes with uncertain gastropod hosts but likely to be freshwater snails (*Macrobilharzia*); (3) two distinct switches of schistosomes from birds to mammals are supported, albeit still with somewhat tenuous support, with one lineage containing the Afro-Asian *Schistosoma* and *Bivitellobilharzia* and the second the North American *Schistosomatium* and *Heterobilharzia* (Ebbs et al., this volume), and (4) a large avian-infecting clade that comprises approximately 17 genus level lineages that might grow further to encompass ~20 genera or more. 

Several schistosome lineages have been retrieved from specific vertebrate groups, suggesting some fidelity to the phylogenetic and/or ecological affinities of their definitive hosts. Some examples include *Macrobilharzia* from cormorants and anhingas, *Anserobilharzia* from geese, *Allobilharzia* from swans, *Trichobilharzia* from anseriforms, *Bivitellobilharzia* mostly from elephants but also known from Asian rhinos, and *Schistosomatium* from rodents. Although some degree of host specificity is evident among schistosomes with respect to their use of definitive hosts, rarely is any schistosome species confined to a single species of definitive hosts. Some schistosome lineages, by contrast harbor species such as *Bilharziella polonica* or *Dendritobilharzia pulverulenta* from birds and *Heterobilharzia americana* and *Schistosoma japonicum* from mammals, which infect distinctly broader spectra of definitive hosts.

**Figure 4:**
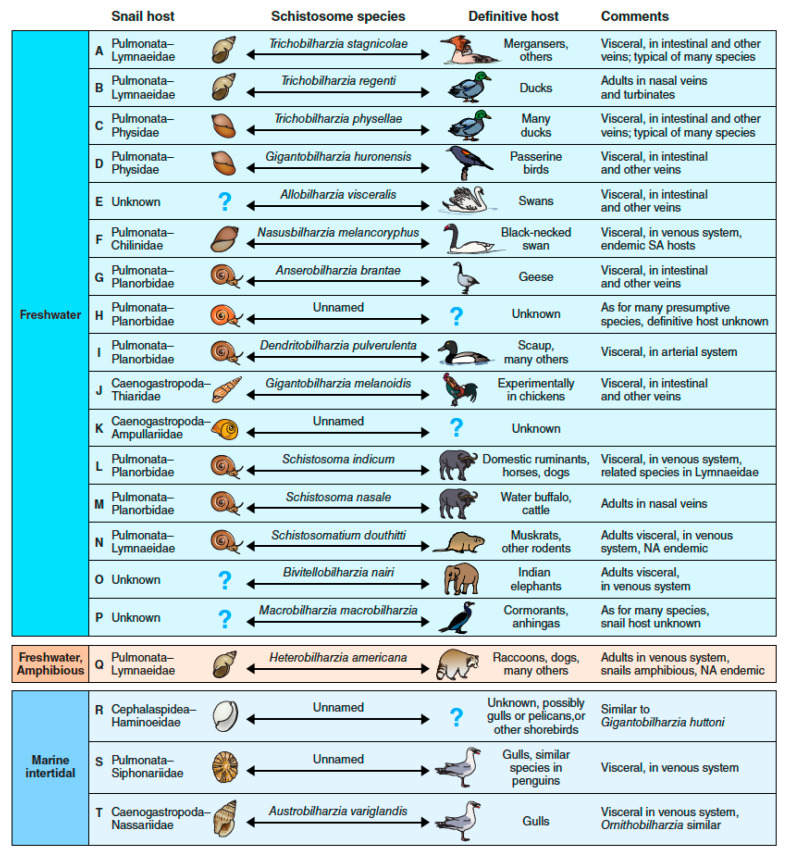
There are several different biological contexts in which swimmer’s itch might occur (see corresponding text for more details). The double headed arrows emphasize the connectedness between the gastropod host and the vertebrate host in any schistosome life cycle that, in more detail, operates as shown in Figure 1 and Figure 5.

As has been discussed before [5], and is also accentuated in Figure 4, as presently known, schistosomes collectively have exploited gastropods from 16 different families in the two most derived lineages of gastropods: the Caenogastropoda and the Heterobranchia [36,46,47]. Caenogastropods ranging from basal to derived representatives have been exploited by schistosomes, whereas amongst heterobranchs, most schistosomes have exploited the relatively derived freshwater Hygrophila lineage. Schistosomes have not, to our knowledge, colonized the three major basal lineages of gastropods (Patellogastro-poda, Vetigastropoda, and Neritomorpha). Interestingly, two related groups of flukes have even broader ranges of intermediate hosts. As far as is known, blood flukes of fishes (Aporocotylidae) exploit caenogastropod and heterobranch gastropods as well as annelids and bivalves as intermediate hosts [48]. Turtle blood flukes (family Spirorchiidae) parasitize annelids, vetigastropods (keyhole limpets) as well as caenogastropods and heterobranchs as intermediate hosts [49].

Somewhat paradoxically, in spite of the collective breadth of gastropods exploited by the schistosome family, individual schistosome species exhibit considerable specificity with respect to their molluscan hosts. A particular species typically infects a single gastropod genus or closely related genera from the same family. In spite of their evident host specificity with respect to gastropods, closely related species are often found in different snail families, suggesting that host switching has been a prominent feature in their diversification [5]. The most basal schistosomes are known from marine caenogastropod snails, but more derived schistosomes largely diversified in freshwater gastropods, including freshwater or amphibious caenogastropods, with most but not all species infecting heterobranchs, particularly freshwater pulmonates. Among the most derived avian schistosome lineages, evidence consistent with host switches from freshwater back to marine gastropods have occurred on at least two occasions [28,29], in each case involving switches into marine heterobranchs (Siphonariida and Cephalaspidea) that are by no means close relatives of the pulmonate heterobranchs [47]. 

Improvement is needed to fully resolve relationships among the schistosome genera (see Ebbs et al., in this volume), and some deeper nodes within the tree still defy confident resolution. For example, the degree of support for two separate origins of mammalian schistosomes might be strengthened by additional mitochondrial or whole genome sequences of representatives of the four genera involved. The phylogenetic position of *Macrobilharzia* might also be clarified by additional information about its host snails and additional sequence data. More attempts to place dates on various events in the schistosome tree have been forthcoming in recent years (see Ebbs et al. for further discussion). It seems reasonable that schistosomes did not appear on the scene until caenogastropods first appeared in the late Silurian and early Devonian about 400 million years ago [50]. Representatives among living schistosome species infect both relatively basal caenogastropod lineages such as Potamididae and Batillariidae and more derived groups such as the Nassariidae [46]. If proto-schistosomes arose from blood flukes of turtles, a more recent origin for Schistosomatidae is suggested, as turtles did not arise until the mid to late Triassic (240–200 mya), with at least one lineage (leatherbacks) displaying characteristics consistent with a degree of endothermy arising less than 100 mya [51]. The molecular clock analysis of Jones et al. [45] retrieved a divergence time of 22.13 mya for the split between *Schistosomatium* and the derived clade of avian schistosomes vs. the mammalian schistosomes *Bivitellobilharzia* and *Schistosoma*, and estimated the origin of *Schistosoma* at about 20 mya, in the Neogene period, somewhat older than estimates of Snyder and Loker [42] based on the fossil record of pomatiopsid snails (16–11.6 mya), the snail hosts for basal *S. japonicum*. Re-sequencing followed by coalescent modeling suggested *Schistosoma mansoni* and *S. rodhaini* diverged about 107–147 thousand years ago [52]. The relatively late appearance of the latter two species, both dependent on *Biomphalaria*, is at least partially explicable by the relative recent colonization (4.3–1.4 mya) of Africa by snails of this genus [53]. One of the imponderables in trying to piece together the evolution of blood flukes, including schistosomes, is their potential representation not only in dinosaurs, but also in prominent groups of mammals with aquatic tendencies such as hippos or proboscideans for which, today, we have very limited representation of what once existed.

## 3. How Much More Diversity Is out There?

We now have better tools than ever to reveal the diversity of schistosomes in objective ways, but it is difficult, specialized work to retrieve the specimens, and often the material obtained is not optimal. Furthermore, how do we know how far we have yet to go with respect to characterizing the available diversity with the thought in mind that the window available for us to do so may be closing? Recent work suggests there is still plenty of novelty to be found: three new genera have been recently described and eight more likely genera await further study and description. Based on 28S sequence data for the 37 avian schistosomes shown in Figure 3, there are 18 named species for which we know both avian and gastropod hosts, 3 named species for which we know only the avian host, 2 unnamed species for which we know both hosts, one unnamed species based on adults only, and 13 unnamed species known from cercariae only. Also, as noted in Figure 2, given that schistosomes with new sequence signatures continue to be found, it seems we are not yet there with respect to getting the full picture of schistosome diversity.

As a reminder of the need be keep an open mind, and to be receptive to surprises in the search for blood fluke diversity, two relatively recent discoveries are noteworthy: (1) of a spirorchiid with annelid rather than gastropod intermediate hosts [49], and (2) of an adult aporocotylid blood fluke (normally found in fishes) from a marine mammal, a dugong [54]. What other fascinating surprises await?

## 4. The Many Paths to Swimmer’s Itch, a Complex Array of Zoonotic Players

The literature about schistosomes causing swimmer’s itch tends to be biased toward species from waterfowl responsible for outbreaks occurring during recreational activities in freshwater lakes in European and North American localities. In reality, as shown in Figure 4, the variety of schistosomes involved in potentially causing dermatitis is surprisingly large with respect to: the phylogenetic diversity of both gastropod and definitive hosts used, the habitats in which their adult worms occur within their definitive hosts, the physical environment in which the life cycle occurs, and how abundant or widely distributed their definitive or intermediate hosts are. In the text below, for each letter indicated in the figure, a brief account follows of the distinctiveness of that particular path to swimmer’s itch.

**A.** The schistosomes and hosts involved fit a familiar pattern (see *T. stagnicolae* in Figure 1), with the adult worms found in visceral veins of waterfowl, eggs passed in the feces, and snails of the family Lymnaeidae serving as intermediate hosts. Prominent examples are *T. stagnicolae* in North America [55,56,57,58,59,60,61], *T. szidati* and *T. franki* in Europe [17,62,63,64,65,66,67,68,69,70], and a species long known from cercariae and swimmer’s itch outbreaks but only recently described from adult specimens and named *T. longicauda* in New Zealand [71]. The snails involved tend to be associated with higher latitudes and are relatively large, which may lead to high cercariae production and, therefore, increased likelihood of being involved in swimmer’s itch cases. [55].

**B.** A similar kind of situation to A, but note the adult worms are found in the nasal mucosa of their waterfowl hosts. They reach this location using a neurotropic migratory route. Exemplified by *Trichobilharzia regenti*, this species may pose particular dangers for immunocompromised humans because the cercariae of this parasite, after penetrating the skin, might escape and begin a neurotropic migration [72,73]. Any of the schistosomes with nasal egg-laying sites deserve consideration in this regard. Another avian schistosome, *Bilharziella polonica*, not known to develop in the nasal mucosa, has also been recovered from the avian nervous system, raising a concern that neurotropic behavior may be more common than previously supposed [74].

**C.** Depending on the schistosome species involved, other families of snails can be involved, in this case snails of the family Physidae, some species of which have been widely distributed around the world [75]. This creates the potential for some avian schistosomes to become cosmopolitan in distribution [75,76]. For instance, the species indicated, *T. physellae*, is indigenous to North America but has recently been reported in Europe, exploiting the introduced physid snail that has established there [77]. Khosravi et al. [78] report a similar cosmopolitan distribution for the marine schistosome *Ornithobilharzia canaliculata*.

**D.** Exemplified is a schistosome exploiting not anatid waterfowl but passerine birds living around aquatic habitats, thus representing a distinctive schistosome colonization of an avian order containing more than half of all bird species. The extent to which this schistosome might be involved in causing dermatitis cases is not well-known but should not be discounted in swimmer’s itch surveys.

**E.** Shown is a distinct schistosome genus with a distinct predilection to infect swans, which often suffer pathological damage as a consequence [79]. In spite of the prominence of the definitive host, the snail host for this schistosome remains unknown.

**F.** This schistosome uses yet a different family of pulmonate snails (Chilinidae), endemic to southern South America [80,81]. It exemplifies a schistosome inherently limited in its geographic distribution by its snail host preference, and is the only known member of the most recently erected genus *Nasusbilharzia* [22]. *Chilina* is an important host genus for avian schistosomes in South America, and co-infections of two species have even been identified from single specimens of *C. dombeyana* [82].

**G.** This schistosome again exemplifies that at the generic level, schistosomes may be confined to a particular group of birds, geese in this case. The abundance of a definitive hosts species such as the Canada goose (*Branta canadensis*) favors this schistosome. Even though the snail host (*Gyraulus*) is tiny (<1 cm in diameter) relative to many schistosome-transmitting snails, because of its local abundance, a population of infected *Gyraulus* snails can still collectively produce enough cercariae to cause a swimmer’s itch outbreak [83,84].

**H.** There are several schistosome taxa known only from their cercariae, including in this example from planorbid snails. For instance, schistosome cercariae recovered from snails such as *Gyraulus* in North America, or *Ceratophallus* or *Biomphalaria* have unknown definitive hosts. Many snail species, not just from the Planorbidae, await further screening. One recent example from North America has revealed a schistosome for the first time from *Planorbella trivolvis* and, in this case, as an example of how a definitive host can be identified it matches sequences recovered from schistosome eggs from the Canada goose [27].

**I.** Whereas schistosome adults are usually dimorphic and live in the venous system, this species produces male and female adults with similar body shapes and lives in the arterial system of diving ducks like scaup, preferably, but can be found in many anseriform hosts [85].

**J.** and **K.** These schistosome serve to remind us that snails of another major gastropod lineage, the Caenogastropoda (instead of just Heterobranchia) can host schistosomes [86,87]. They are often poorly known, with unknown definitive hosts and uncertain roles in causing dermatitis. For many such cercariae, the corresponding adult stages are unknown; consequently they have not been formally named.

**L.** Whereas the schistosomes mentioned thus far have avian or unknown definitive hosts, cercariae of *Schistosoma* species normally developing in the liver and intestinal veins of ruminants can also cause swimmer’s itch [88,89]. As noted above, human-infecting species can also cause dermatitis. Perhaps somewhat more perplexing is that some ruminant schistosomes such as *S. bovis* are very common in Africa, and snail infections are commonly recovered, but reported cases of swimmer’s itch due to this species are rare. It is not clear why: it may simply reflect a lack of reporting.

**M.** This mammalian schistosome is unusual in developing in nasal rather than visceral veins of cattle and buffaloes [90]. Whether cercariae of this species pose particular neurological dangers to its normal hosts or to humans when encountered deserves further study [91]. It should be considered that it might also have neurotropic tendencies such as *T. regenti*.

**N.** This schistosome is peculiar for being endemic to North America, developing in rodents such as muskrats, and producing cercariae that emerge from snails at night [92]. Whether these cercariae actually are implicated in human swimmer’s itch cases is debatable because the life cycle occurs in marshy habitats often not frequented by people and the cercariae emerge at night, but cercariae certainly cause swimmer’s itch when placed on the skin [93].

**O.** Both Asian and African elephants, and at least two of the five extant species of rhinos, harbor schistosomes, and although the snail hosts for them are not known [23], the fact that elephant handlers are known to experience swimmer’s itch after contact with streams in which they regularly water and bathe elephants is suggestive of the possibility that cercariae of elephant schistosomes are involved. All matters related to the unknown natural intermediate hosts of these intriguing schistosomes require further investigation.

**P.** In this case, as for several additional schistosome species, the snail host is not yet known with certainty [94], even as to whether it is freshwater, estuarine, or marine in habitat. This unusually large schistosome is found only in cormorants and anhingas, and its role in causing swimmer’s itch is unknown. Some schistosomes infect snails found in habitats transitional between freshwater and land (as on muddy or wet banks or in rice paddies).

**Q.** This North American schistosome infects *Galba* snails (Lymnaeidae) living on muddy surfaces, but the cercariae make their way to water and can cause swimmer’s itch in people [95]. The human-infecting *Schistosoma japonicum* developing in amphibious *Oncomelania* snails is another species living in transitional habitats that traditionally caused dermatitis called paddy itch. Finally, some schistosomes living in marine or brackish water snails can also cause swimmer’s itch.

**R.** In this case, marine heterobranch bubble snails (Haminoea) living in the intertidal region have been implicated in causing swimmer’s itch outbreaks. The definitive hosts are not known with certainty but are likely to be gulls or their relatives, or pelicans. An exotic Haminoea has been introduced on the Pacific coast of North America and been implicated in itch outbreaks [96].

**S.** This schistosome is noteworthy for exemplifying species able to infect a quite different kind of gastropod—an air-breathing pulmonate intertidal gastropod [29]. Gulls and possibly penguins may play a role as definitive hosts. Whether this species actually causes dermatitis cases in people should not be ruled out.

**T.** Finally, yet a different lineage of snails is involved here, a burrowing mud-inhabiting caenogastropod intertidal snail that transmits a gull schistosome implicated in causing what is known as clam-digger’s itch. *Ilyanassa obsoleta*, the Atlantic or eastern mudsnail represents another invasive marine snail that has colonized habitats on the Pacific coast of North America and has been implicated in causing swimmer’s itch outbreaks on both coasts [97]. Representatives of *Austrobilharzia* have been recovered from both basal caenogastropods such as Potamiididae, or more derived representatives such as *I. obsoleta*.

## 5. Swimmer’s Itch—A One Health Perspective

The concept of One Health, as defined by the U.S. Centers for Disease Control, is “a collaborative, multisectoral, and transdisciplinary approach—working at the local, regional, national, and global levels—with the goal of achieving optimal health outcomes recognizing the interconnection between people, animals, plants, and their shared environment.” As noted above, swimmer’s itch is an example of a zoonosis, and insofar as swimmer’s itch is a condition experienced by humans (but other non-host species also surely experience this as well) and is one that features many biological connections with various animal species and their environments, the One Health paradigm seems also particularly relevant. Given changes in aquatic habitats, and waxing or waning of the various species involved, in some contexts swimmer’s itch can be considered an emerging or re-emerging disease as well [98]. 

**Figure 5:**
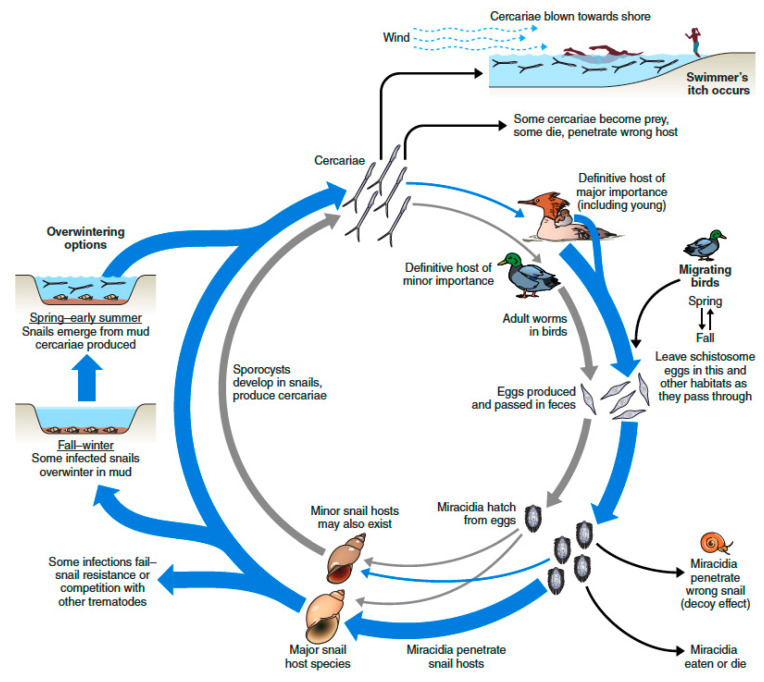
An alternative version of the life cycle of *Trichobilharzia stagnicolae* shown in Figure 1, taking into account at least some of the myriads of circumstances that might impact the parasite and its tendency to cause swimmer’s itch outbreaks. See text for discussion.

The emphasis here is on the growing realization that the relatively straight-forward depiction of the life cycles or schistosome species involved (Figure 1), although an essential baseline for understanding the underlying biology, is one that can also be reconsidered in a broader, more inclusive way (Figure 5).

At the core of the diagram in Figure 5 is the essential life cycle shown in Figure 1 involving common mergansers (*Mergus merganser*) as definitive hosts, and *Stagnicola emarginata* as the most important snail host. The discussion below mentions *T. stagnicolae* as an example, but the various circumstances discussed are often generalizable to many, if not all, schistosome species. 

Figure 5 acknowledges the possibility that other host species may be involved, and even though they might be considered of minor overall importance, could provide infective stages (miracidia or cercariae) at key times during the year, or potentially provide some buffering capacity for the parasite should one of its major host species decline [20,99]. This might occur with *T. stagnicolae*, for example, if migratory bird species with only a transitory presence [20], or an unknown local bird species, perhaps even domestic ducks, were actually releasing schistosome eggs at critical times to perpetuate the schistosome’s life cycle. Similarly with respect to snail hosts, if oligotrophic lakes in the northern U.S. or Canada warmed or experienced heavy nutrient loads, populations of *S. emarginata* might crash and possibly be replaced by more tolerant species such as *Physa acuta* [100,101], which might then come to support a very different set of avian schistosome species (e.g. *Trichobilharzia querquedulae* or *T. physellae*), still capable of potentially causing itch outbreaks but with quite different definitive hosts involved, and very different seasonal transmission dynamics. Populations of the host snail might falter if exotic molluscs such as zebra mussels, New Zealand mud snails, or Chinese mystery snails (*Cipangopaludina chinensis*) were introduced [102,103,104,105], or if predator populations were altered [106]. To generalize, it seems very likely that predicted climate or biotic changes will cause changes in snail species composition with consequent changes in the schistosome species composition, which might result in very different kinds of swimmer’s itch concerns. 

The fates of miracidia and cercariae released into the water are many, and often end in failure. They may die if they fail to find a suitable host before their limited energy reserves run out. Onshore winds might push cercariae or miracidia into shallow edge waters [57,107,108] where appropriate host species are typically not found (but toe-dipping humans are found). Miracidia and cercariae may become prey items for aquatic invertebrate predators [109], the latter possibly including symbiotic oligochaetes living on the snails themselves [110]. Another unproductive fate is that they might encounter what is often referred to as the dilution effect [111], or a similar term often used with respect to miracidia and snails, the decoy effect [112]: miracidia or cercariae may penetrate inappropriate hosts and then die. In other words, the transmission of the disease-causing schistosome could be diminished or diluted in biologically diverse habitats when they enter hosts that effectively serve as unproductive sinks for them. From the schistosome’s point of view, any cercariae that might penetrate and then perish in the skin of humans or other non-host species are a dead end with respect to completion of its life cycle, scant consolation for the person suffering from swimmer’s itch. 

Recently, it has been noted that miracidia of itch-causing *T. regenti* and *T. szidati* are attracted to water conditioned by non-host snails [113], and that lakes containing the schistosome-resistant invasive snail *Potamopyrgus antipodarum* had lower prevalence of trematode infections in *L. stagnalis*, the species normally transmitting *T. szidati* [18]. This has prompted a suggestion that *P. antipodarum* might have a role to play in defending European lakes from itch outbreaks [18,114]. A similar role for other species acting as biological diluents for itch-causing cercariae has also been proposed [115]. 

Another hazard with respect to the *T. stagnicolae* miracidia shown in Figure 5 is that they might be successful in penetrating an individual *S. emarginata* snail, only to find it has been previously colonized by larvae of a competing trematode species that actively attack and consume, or otherwise suppress, the schistosome’s intramolluscan development. This phenomenon is real for several schistosome species vying with other trematode species for access to a compatible snail host [116]. The abundance of these competing trematodes will be influenced by the abundance of the various second intermediate and definitive hosts upon which they depend, another way in which biotic complexity can dampen transmission of itch-causing species. As another hazard, a schistosome may enter a snail only to have it mount effective defense responses and either kill it or prevent it from developing [117]. Once a successful schistosome infection has been initiated in the snail host, competitor trematodes might later colonize the snail and outcompete the schistosome, the snail might be eaten by a predator, or the snail might die of natural causes within the approximately one month period needed for schistosomes to produce cercariae [118]. Schistosome-infected snails may engage in behavioral responses such as moving into favorable thermal regimes to minimize their likelihood of mortality [119], but it is commonly the case that infected snails have higher mortality rates than uninfected snails [118].

As indicated on the left side of Figure 5, with *T. stagnicolae* and other schistosomes living in temperate water bodies, infected snails can enter the bottom sediments of their habitats and overwinter [120,121,122]. In the following spring, these snails emerge and, after a period of warming temperatures needed to allow sporocysts to re-commence cercariae production [123,124], can begin to release cercariae in the water again. This has potentially very important consequences because cercariae from the previous year’s infections are available to infect definitive hosts, particularly the susceptible young of the year birds, or migrating birds passing through [20]. Thus, critically-important transmission episodes likely occur long before the water is warm enough to entice human water contact. Young birds with new infections, or older resident or migrating birds with old infections, might all potentially produce eggs that can seed a new generation of snail infections [20]. The origins of the schistosome infections in snails responsible for mid- to late-summer outbreaks of itch remains something of a black box. 

In general, as shown on the right of the figure, the role of avian migrations in the life cycles of itch-causing schistosomes also remains somewhat perplexing. For instance, one possibility is that migrating hosts widely broadcast schistosome eggs across many habitats on both legs of their migratory journeys, and also continually pick up new infections at stop-over sites. This version would be favored if the essential snail host species (such as *Physa*) were ubiquitously common, and adult schistosomes were long-lived or new worms successfully colonized already-infected hosts. This would seem to create the opportunity for panmictic schistosome populations, and some evidence, as for the cosmopolitan *T. querquedulae* with little population structure [76], supports this notion, as do studies of avian schistosomes using marine gastropods [29,78]. Another possibility is that the input of schistosome eggs of migrating subadults or adults is not as pronounced, and that snail infections originate mostly from resident birds and their progeny, placing considerable emphasis on the vulnerability of young birds to infection. Some results from carefully monitored experimental infections suggest that transmission by ducks of *T. ocellata*, now considered to be *T. szidati* [125], might be fleeting, as worms developed to maturity rapidly (7 days) and had ceased egg-laying by 21 days [126]. The authors remarked this rapid development was likely an adaptation to migratory hosts, insuring infection of snail hosts on the breeding grounds. Of course, it is possible that a middle course between these extremes is followed. One possibility of interest is that adult male worms may live longer than females, accounting for their preponderance in natural infections [127], and they encounter new crops of females as their host migrates from one habitat to another. Usually absent from the story of how the life cycle of avian itch-causing schistosomes functions is an indication of whether birds develop strong immunity to reinfection. Some experimental evidence, again with *T. szidati*, suggests ducks do develop strong resistance to challenge with homologous species [128,129,130]. This may vary with the schistosome species under consideration, and as a function of how closely matched the schistosome is to various species of avian hosts.

To summarize this section, it is obvious that the ecological context in which itch-causing schistosomes live is complex, with sometimes very surprising impacts from snail species that might be native or possibly of recent exotic origin. The quantitative inputs of resident and migratory hosts to these life cycles remain to be determined. Although it might seem that all the vicissitudes experienced by schistosomes would overwhelm them and doom them to failure, it must also be appreciated that schistosomes are capable of prodigious reproductive feats. For example, individuals of the large snail *L. stagnalis* infected with *T. szidati* can produce up to 29,560 cercariae per day, with an average of 2,621 cercariae produced per day. The aggregate production of cercariae over the snail’s lifespan has been estimated to be comparable to, or even exceed the snail’s own body mass, and collectively the cercariae produced within a population of *L. stagnalis* over the course of a year is estimated to exceed the weight of an elephant [131]. Clearly, schistosomes have their ways of coping. 

There is also the overriding importance of the aquatic environment itself and how that environment responds to impending changes, not just with respect to schistosomes but also for the many hosts that support the parasites. For instance, there has been a recent worrisome global increase in highly pathogenic variants of avian influenza HPAI A(H5) that, in addition to devastating domestic poultry flocks, also infects many prominent groups of definitive hosts for avian schistosomes such as ducks, geese, gulls, terns and swans as well as shorebirds [132]. The knock-on impacts of this virus on transmission of avian schistosomes, or potentially of virus-schistosome coinfections [133] on avian definitive hosts, are largely unknown and await further study. Developments such as these emphasize the value of backing out, taking the broader One Health perspective, and enlisting the expertise of local grassroots stakeholders [134] and a broad spectrum of interdisciplinary-minded scientists to find a way forward to achieve a greater understanding of the place of itch-causing schistosomes in nature. 

## 6. Monitoring Itch-Causing Parasites in Natural Habitats—Some Pros and Cons of Different Methods

One aspect of the biology of dermatitis-causing schistosomes that the public most cares about is where they occur and when, such that they can attempt to avoid becoming victims of swimmer’s itch. Traditionally, this has leaned heavily on collection of samples of suspect aquatic gastropods, which are then isolated in small containers to see if they release, or “shed”, cercariae into the water (see Born-Torrijos et al. [117] for a good discussion). The cercariae are then examined microscopically, and those identified generically as schistosomes are enumerated and subject to further identification. Microscopical examination of the schistosome cercariae recovered is almost never sufficient to correctly identify them to species, requiring follow up application of sequencing techniques for more objective identification. Sequences for commonly used reference barcode genes, such as *cox1* or ITS2, are obtained and compared to available databases such as GenBank. The shedding method is used the world over, whether to survey for human or non-human schistosomes [135].

Alternative surveillance approaches for assessing the presence of cercariae in natural water bodies are available [56,108,136]. Many rely on the collection of water samples that might contain cercariae, eggs or miracidia, or schistosome DNA (collectively called environmental or eDNA samples here for convenience), followed by concentration of the sample and subjecting it to DNA extraction. Then, amplification follows using various techniques such as conventional PCR, qPCR, recombinase polymerase amplification (RPA) or loop-mediated isothermal analysis (LAMP) in conjunction with various kinds of primers specific for schistosomes or for particular species of schistosomes (and possibly snails too). Read-outs of the results vary with the technique and range, from identifying amplified bands on gels to detecting increasing fluorescence in real time using a fluorometer, or to direct visualization of amplification products in a reaction tube. The latter molecularly-based approaches allow for collection of many filtered water samples, avoids the need to collect and screen snails, and offers a way forward for identification of one or more schistosome species recovered [56].

Pros and cons of these and other methods have been discussed [135,137]. For the purposes of documenting biodiversity, having the actual sample of the infected gastropod and the schistosome cercariae it is shedding provide enduring samples for natural history collections. These tangible specimens can be used to verify identities and to undertake any number of future studies, including provision of complete genome sequences for both snail and cercariae. eDNA samples can likewise be archived in collections and provide a valuable snapshot of a broad variety of DNA in the water at the time and location of the sample. Environmental DNA samples leave somewhat ambiguous the source of the DNA being amplified: live or dead miracidia or cercariae, or fragmented schistosome DNA, although usually it is cercariae from the water that provide the DNA in the sample [108]. Depending on the context, it may be less important to know the exact life cycle stages from which the eDNA is derived, as the more important consideration might be simply to determine whether active transmission is occurring at a particular site. A positive eDNA sample would be suggestive of the recent presence/transmission of the parasite. Samples of eDNA are complex mixtures of DNA which may be in various degrees of degradation and so are less likely to provide a great deal of long read length schistosome DNA for use in additional kinds of studies. Such samples do not identify the gastropod host species that supported cercariae production, although DNA from a variety of gastropod species may also be present in the sample to help facilitate such a determination. 

Collection of an infected snail can provide explicit information about the time and location of the source of itch-causing cercariae but says little about where the cercariae may end up. Snail collection is labor-intensive, may require specialized collection equipment such as dredges and boats, creates the possibility that the collectors may themselves be exposed to infection, and the expertise required to differentiate among the many types of cercariae, most of which are not schistosomes, is not trivial. It is often desirable to reisolate and shed collected snails after a week or two to identify infections that were not fully developed at the time the snail was first collected. The shedding method underestimates prevalence of infection in general, including double infections [138].

In contrast, eDNA samples provide information about where schistosome eDNA (probably from cercariae) is at a specific point and time which is important information for people worried about swimmer’s itch but says less specifically about where the shedding snails are found. One of the intriguing aspects of eDNA samples is that due to the mixing of water, they integrate schistosome signals over time and space, and this is helpful with respect to increasing the chances of determining if schistosomes are present, and if so, of what species. Additionally, because DNA from schistosomes may persist after their death, eDNA paints a somewhat impressionistic view of the relative risks at specific times and places. Positive eDNA results from samples containing cercariae dead for 1 to 3 weeks have been obtained [139,140], but rates of degradation of signals in natural habitats may be faster [140].

One interesting and pragmatic approach being considered for monitoring the presence of human-infecting schistosomes in aquatic habitats is to collect and pool specimens of vector snails in a single water container, allow the snails to shed cercariae in the water, and then test the water using an eDNA based detection technique [141]. This hybrid method greatly reduces the labor and expense of isolating individual snails and examining them for infection by either microscopical or molecular methods and provides a water sample potentially amenable to successful extraction and amplification of a schistosome signal. It is less useful for pinpointing where cercariae may end up in a particular habitat.

In our view, collection of environmental water samples and associated eDNA will be the method of choice for monitoring the presence of schistosome infections in waterbodies: large numbers of samples can be collected, samples can be rapidly analyzed, species-specific primers can be devised, and, importantly, the samples can offer a quantitative measure of the amount of schistosome DNA present, but caveats occur in interpreting the data. Additionally, due to an increased emphasis on diagnostics as part of the control and elimination efforts for human schistosomiasis, and with encouragement from the World Health Organization, a number of different molecularly-based platforms for schistosome nucleic acid detection are being developed, tested and streamlined to be faster, cheaper, and to minimize dependence on expensive lab equipment (e.g. [142]. The ultimate goal is to develop point-of-need techniques that can be administered by personnel with minimal specialized training. Such an outcome would also be highly desirable with respect to communities interested in monitoring the relative risks of swimmer’s itch outbreaks in local waterbodies. Here it should be noted that the qPCR-based studies of detection of swimmer’s itch in Michigan lakes have provided an important proof of principle for the value of molecular monitoring of schistosomes in natural habitats [56,108,134].

Some collection of actual *specimens* of infected snails and the cercariae they are producing are also needed to verify the identity of infected snails and to have additional biological materials for vouchers sent to permanent repositories for further study. Indeed, the recently-released WHO guidelines on control and elimination of human schistosomiasis [143] call for using two methods to monitor the presence of schistosomes in snail populations to ensure specificity and sensitivity, one being snail shedding and the second a molecularly-based diagnostic technique such as qPCR, LAMP or RPA assays. 

## 7. Controlling, or Should We Say Managing, Swimmer’s Itch—The Need for Effective Yet Specific and Environmentally Acceptable Solutions

As noted in the review by Soldánová et al. [17], efforts to mitigate the adverse effects of swimmer’s itch need to carefully weigh the economic and environmental costs against the risks and harm posed by what is an unpleasant but not really dangerous disease, although the risks posed by some non-human schistosomes may be greater than we generally appreciate. Furthermore, swimmer’s itch parasites occur in locations ranging from pristine natural habitats to heavily used recreational areas, so any management efforts are likely to be context-dependent. For understandable reasons, the thoughts surrounding swimmer’s itch control are in many ways similar to those considered over the years for control of human schistosomiasis, but again, the cost-benefit analyses and background contexts are often quite different. 

A variety of solutions have been sought to prevent swimmer’s itch outbreaks, and it is fair to say that none have proven ideal or widely applicable. Soldánová et al. [17] have provided an excellent overview of several possible preventive measures indicating specific parts of the schistosome life cycle targeted, mode of action, and estimated effectiveness and environmental impacts. Here we add some additional comments regarding recent approaches.

One common approach is to target the snails responsible for producing the cercariae that cause swimmer’s itch. With respect to human schistosomiasis, it has been argued that control programs targeting snails are more likely to be effective than those that do not [144], and a variety of inventive approaches have been suggested [145]. Molluscicidal chemicals such as copper sulfate or niclosamide have often been considered for this purpose. Focal application of molluscicides can be an effective component of integrated human schistosomiasis control programs [146]. Molluscicides, though, are relatively indiscriminate with respect to the molluscs they kill, can be costly, and might affect other animals in the system, some of which might contribute some measure of natural control. As shown recently, although molluscicides might kill target snails in the treated area, itch-causing cercariae from outside the treatment area may still drift into the treated area, thereby defeating the purpose of focalized application [147]. Unless much more target gastropod-specific, rapidly degrading and environmentally safe molluscicides are developed, this approach will be increasingly hard to justify as a modern swimmer’s itch control method. 

The use of biological control measures such as predators of snails or cercariae, snails that compete with itch-transmitting snail populations, or snails that serve as decoys for schistosome miracidia have long been contemplated [114,148,149]. The case presented by the accidental introduction of the New Zealand mud snail *Potamopyrgus antipodarum* into Polish lakes is of particular interest due to apparent effects in lowering rates of infection in *Lymnaea stagnalis* of *T. szidati* by exerting a decoy or disorienting effect on miracidia [114]. Laboratory results suggest similar effects on miracidia of *T. regenti* miracidia attempting to penetrate *Radix balthica* [18]. Both of these species are high on the list of causative agents of swimmer’s itch that would be desirable to control, and the authors are to be applauded for revealing a potent mechanism disfavoring their transmission. Any thoughts though of deliberate introductions of *P. antipodarum* to control transmission of these itch-causing schistosomes, even into limited recreational areas, should engage a broad community of aquatic biologists and should be based on longer term data that might reveal accommodations made by the schistosomes involved to overcome any inhibitory effects of the introduced snail. In the meantime, the introduced snails could have far-reaching effects on lake environments that might prove impossible to reverse.

Another basic approach to swimmer’s itch control involves attacking adult worms inhabiting definitive hosts. Efforts have been made to treat wild birds with praziquantel as a means of killing adult worms, thereby preventing the emission of schistosome eggs, in turn limiting the number of cercariae-producing snail infections [150,151]. Extensive programs of treatment of humans with praziquantel are generally successful for lowering the prevalence or intensity of infection, but it is generally acknowledged these programs do not achieve transmission control [152]. Programs targeting wild populations for treatment seem even less likely to achieve control, but this approach could prove useful if domestic birds were the primary source of itch outbreaks.

In some locations, as in lakes in northern Michigan where dermatitis outbreaks often involve the life cycle shown in Figure 1 and Figure 5, programs have been developed for selective capture and relocation of particular definitive host species, such as common mergansers, deemed to be critical for the schistosomes’ ability to produce eggs and thus ensure maintenance of infected snail populations. Positive results of capture and relocation of mergansers on snail infections have been noted by some [153], but others have discounted the value of this approach, in part because migratory birds may also contribute to snail infections [20]. Manipulating and relocating natural populations of waterfowl for the sake of swimmer’s itch control is both labor-intensive and can invite concern from elements of the public who want their lake habitats left undisturbed. On the other hand, the knowledge that local breeding waterfowl may be driving local schistosome populations, and that their removal can dramatically lower snail infection rates [153], can lead local lakefront owners to resort to illegal hunting or disturbance of nests.

Another approach awaiting further testing is to use floating baffles as physical barriers that prevent cercariae, which are usually found in the upper layer of the water column, from entering recreational swimming areas [154]. Further testing is required to improve designs to actually remove accumulated cercariae from the water lest the cercariae concentrated by such baffles pose dangers to people swimming near or just outside the baffles. This approach also raises reasonable concerns about aesthetics, potential degradation of plastics originally designed for short term deployment, storm damage and debris, and right of way issues for the public.

Given the difficulties in exercising control efforts that are feasible, cost effective and environmentally acceptable, barring the as yet unknown magic bullet that might selectively kill schistosome infections in definitive hosts or snails without generating any other collateral damage, the ways forward may be to manage the problem by responsibly preventing human exposure in the first place [17,107,108,154,155]. Some approaches are: (1) improve overall education about the underlying biology of swimmer’s itch and post signs that clearly mark areas known to have itch outbreaks; (2) put more effort into improving the ability to screen particular areas heavily used for recreation for the presence of cercariae using eDNA approaches to learn when and where it is safest to avoid contact with cercariae; (3) be mindful of the ability of prevailing wind to concentrate cercariae in particular locations and try to design swimming areas that avoid these locations; (4) time swimming activities in the late afternoon when cercariae are least likely to be in the water; and (5) wear swim clothing that can protect the skin from penetration by itch-causing cercariae. Development of creams that can kill cercariae or prevent their penetration of skin yet are not easily washed off have been sought, evaluated and some shown to be promising (e.g. [156], and further development should be encouraged. Such topical treatments should avoid use of ingredients potentially toxic in aquatic environments, including microplastics. Note that all of these measures appropriately tend to put the onus for control more on the people who are using habitats for recreation, not on the habitat itself.

It is one thing to devise methods to control or manage swimmer’s itch in affluent, recreation-oriented contexts, but quite another to protect workers in developing countries who may regularly suffer from itch outbreaks from daily activities such as water collection or rice planting. Meaningful control in such contexts for both human-infecting and itch-causing schistosomes remains an elusive goal. Here though, rather than ending this section on a flat note, we must remain mindful that the tools and knowledge base available for inquisitive new generations of problem-solving oriented biologists have never been better. All kinds of innovative approaches to selective and specific control of pests or parasites are exciting to contemplate. These range from use of CRISPR-facilitated gene drives for engineering the spread of schistosome resistance genes in hosts [157] to discovery and exploitation of symbionts ranging from viruses [158] to metazoans that target schistosome larval stages in snails or adult worms in birds or mammals.

## 8. Outlook and Priorities for Future Work

There has been considerable recent progress with respect to revealing the full extent of schistosome species diversity and identifying the considerable variety of life cycle variations. There is clearly more to do, and there is some urgency in this endeavor: Loss of snail or definitive host diversity will likely mean loss of schistosome diversity. Unfortunately, a rising tide of regulatory requirements will make it increasingly difficult to procure the necessary specimens. Documenting and preserving the biodiversity as voucher specimens, preferably collected with additional genetic and morphological study in mind, in well-established natural history collections, is more important than ever.

A goal to be sought in the near future is to dramatically increase the amount of genome information available for all schistosomes, including representatives from across the taxonomic spectrum. Currently only 3 of the 17 described genera are represented by whole genome sequence data. In the era of low-cost genome sequencing, this should be a priority, especially for species that are rare and in danger of being lost. The advantages of having a greater representation of the overall genetic diversity of schistosomes are bound to be many and will include clues as to how life cycles are orchestrated [159], why some species have broader host spectra than others [160,161], how host shifts might have been undertaken [162,163], how genetically diverse particular species are [164,165], and how we might reveal targets that would make their management more likely [166].

One of the most glaring of shortcomings pertaining to avian schistosomes is the relative shortage of experimental infections of natural definitive hosts, complete with scheduled necropsies and histology to gain a greater understanding of several basic attributes of their biology, including: (1) which host species can or cannot support complete development of worms to patency. That is, how host-specific are the various schistosome species? (2) how long does it take worms to achieve patency? (3) How long do the two sexes survive and females continue to produce eggs? (4) Can hosts that have been exposed to one round of infection be successfully reinfected or do they acquire immunity, and if so, does this immunity wane? (5) Can co-infections with multiple schistosome species be readily established in birds, or are there significant cross-reactions? Answers to these questions will help resolve many of the uncertainties noted above regarding the annual dynamics of transmission. Given the concerns posed by avian schistosomes with neurotropic tendencies, such studies are also relevant to learn if species not suspected of migrating within the nervous system, such as *B. polonica* [74] or others [167], might actually do so more than expected. 

Another goal is to gain more explicit information about what species of schistosomes, snails and definitive hosts are most likely to be involved in actual swimmer’s itch outbreaks, and why. Some schistosomes such as *B. polonica* seem not to cause dermatitis in human volunteers [168], or appear to be less frequently successful in penetrating human skin (Anderson et al., this volume). For some schistosomes, some people react strongly to cercariae placed on their skin, whereas others show no response to the same batch of cercariae. Some snails, such as *Stagnicola palustris* in European waters are not particularly good hosts for avian schistosomes; what does this or other refractory species have to tell us? Given that it is not uncommon to have multiple species of schistosomes and their cercariae in some locations, which species is actually producing the lesions on people’s skin? Are there relatively non-invasive ways to sample lesions on skin followed by DNA sequence analysis to specifically identify the schistosome species involved in causing lesions? 

Among the great uncertainties that lie ahead with respect to distribution, abundance and potential extinctions or changes in relative abundances of itch-causing schistosome species will be the many anticipated influences of climate change. In general, it seems logical to think that increased temperatures will speed up schistosome development times and increase cercariae production in snails and favor transmission [16,169,170]. Warming temperatures in places such as Denmark may already be responsible for increases in dermatitis noted there [171]. These effects might be offset by higher rates of mortality for both cercariae released from infected snails [171] and of infected snails [172]. As noted by Żbikowska and Marszewska [119], infected snails may seek lower temperatures as a way to reduce their mortality rates following infection, also lowering their rate of cercariae production in the process. Global warming is likely to lower thermal barriers to colonization in some lakes, and thus may favor changes in the composition of species involved in dermatitis transmission or introduce species such as *P. antipodarum* that might serve as decoys and lower transmission [173]. Warming may also increase eutrophication rates, which are generally considered to be a factor favoring dermatitis outbreaks [16,174]. Increasing pollution by heavy metals such as lead may have negative effects on bird populations and the populations of parasites they harbor [74]. Clearly there will be much to unpack with respect to climate change and sustained long-term studies of particular habitats prone to outbreaks, from a variety of biomes, would be an ideal way to address this issue. The One Health approach discussed above provides a framework from which to consider the impacts of climate change. 

## 9. Increased Funding Opportunities for Swimmer’s Itch Research?

The study of swimmer’s itch has long fallen into cracks between the priorities of different funding agencies—not biomedical enough for some funding agencies, or too biomedical for others. However, given the growing emphasis on disease ecology, more encompassing conceptual frameworks as presented by One Health and well-exemplified by swimmer’s itch, examples of how swimmer’s itch work can inform human schistosomiasis control, and some of the unpleasant environmental changes that lie ahead that will require action, the time seems ripe for international collaborative efforts that take full advantage of and build from the considerable new body of accomplishment relating to swimmer’s itch. 

As noted previously, the health consequences and costs of swimmer’s itch are difficult to quantify, and are probably of overall minor significance, but may be greater than generally realized if neurotropic schistosomes are involved or if we had more epidemiological data to consider from impoverished places where exposure to swimmer’s itch is a regular and unavoidable occurrence. Assuming developed countries continue to enjoy high standards of public health, a case of swimmer’s itch might be the only exposure to helminths and their antigens that many people from such countries will ever experience. How (repeated) short-term exposures to swimmer’s itch schistosomes might affect overall maturation and responsiveness of the human immune response is deserving of investigation [175].

Although justifying larger scale collaborative efforts of swimmer’s itch using just the criterion of its health consequences or impact on local economies may continue to prove difficult, a more compelling justification might be as a model of a complex, multi-faceted zoonotic disease that serves as a bellwether for the condition of aquatic habitats and the many organisms that inhabit them. Coupled with continued efforts to reveal the extent of schistosome diversity, long-term collaborative studies involving parasitologists, malacologists, ornithologists or mammalogists, immunologists, population geneticists, aquatic ecologists, disease ecologists, and infectious disease modelers devoted to better understanding cycles of transmission and how they will respond to impending environmental changes, offers a compelling way forward.

## 10. Conclusions

Our increasing knowledge of the diversity of avian and non-human mammalian schistosomes, including some representation of samples from all continents except Antarctica, coupled with the ever-expanding and formidable knowledge base for the human-infecting schistosomes, combine to make the Schistosomatidae the best known of the approximately 80 families of digenetic trematodes. As such, the schistosomes serve as an important comparative model system to provide unique perspectives on questions ranging from molecular to evolutionary or ecological scales. Because of the many ways their complex and diverse life cycles interface with biological and physical environments, schistosomes can help us gauge the impacts of climate change, exotic species introductions, and habitat degradation. Although schistosome life cycles have often proven to be remarkably resilient to perturbation, in some instructive cases they have collapsed when key host species have disappeared [176], which may become a common theme in coming years. The simplification of Earth’s aquatic ecosystems may come to favor particular subsets of definitive and intermediate hosts most resilient to perturbation, with resulting great changes in the distributions and health threats posed by various species of schistosomes. Continued discovery and documentation of schistosome diversity will help us better comprehend and mitigate the adverse changes that lie ahead.
